# Silver Nanoparticles and Their Therapeutic Applications in Endodontics: A Narrative Review

**DOI:** 10.3390/pharmaceutics15030715

**Published:** 2023-02-21

**Authors:** Farzaneh Afkhami, Parisa Forghan, James L. Gutmann, Anil Kishen

**Affiliations:** 1 Department of Endodontics, School of Dentistry, Tehran University of Medical Sciences, Tehran 1439955991, Iran; 2 School of Dentistry, Tehran University of Medical Sciences, Tehran 1894787545, Iran; 3 Department of Endodontics, College of Dentistry, Texas A&M University, Dallas, TX 75246, USA; 4 Faculty of Dentistry, University of Toronto, Toronto, ON M5G 1G6, Canada

**Keywords:** endodontics, root canal therapy, silver nanoparticles, metal nanoparticles, nanoparticles

## Abstract

The efficient elimination of microorganisms and their byproducts from infected root canals is compromised by the limitations in conventional root canal disinfection strategies and antimicrobials. Silver nanoparticles (AgNPs) are advantageous for root canal disinfection, mainly due to their wide-spectrum anti-microbial activity. Compared to other commonly used nanoparticulate antibacterials, AgNPs have acceptable antibacterial properties and relatively low cytotoxicity. Owing to their nano-scale, AgNPs penetrate deeper into the complexities of the root canal systems and dentinal tubules, as well as enhancing the antibacterial properties of endodontic irrigants and sealers. AgNPs gradually increase the dentin hardness in endodontically treated teeth and promote antibacterial properties when used as a carrier for intracanal medication. The unique properties of AgNPs make them an ideal additive for different endodontic biomaterials. However, the possible side effects of AgNPs, such as cytotoxicity and tooth discoloration potential, merits further research.

## 1. Introduction

**Figure 1:**
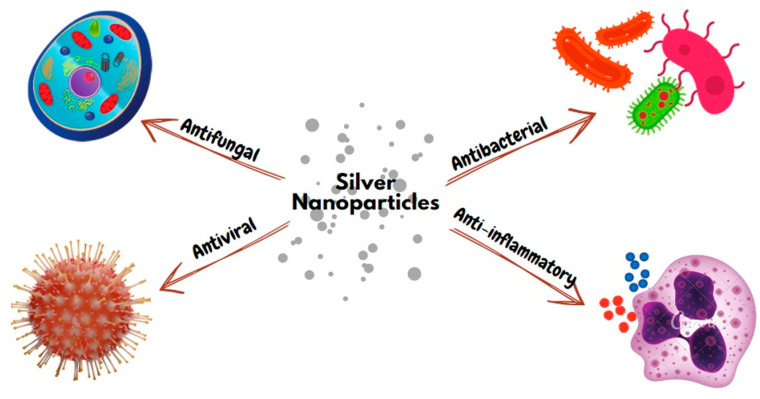
Biological activities of silver nanoparticles.

Silver nanoparticles (AgNPs) are made of pure crystalline silver with dimensions ranging from 1–100 nm. They have recently gained popularity for a wide range of biomedical applications because of their enhanced and unique physicochemical properties, such as smaller particle size, higher surface area, and quantum confinement effects, among others, compared with bulk or powder material [1,2]. AgNPs constitute 56% of all the nanoparticles worldwide [3]. In dentistry, AgNPs are used in the fields of endodontics, restorative dentistry, orthodontics, implantology, prosthodontics, and periodontics [4]. They have been primarily used for disinfection, prophylaxis, and prevention of oral infections due to their favorable antimicrobial properties [5]. This article provides a comprehensive review on the antibacterial, antiviral, antifungal, and anti-inflammatory properties of AgNPs and their safety for clinical applications (Figure 1). Different endodontic applications of AgNPs in root canal irrigants, intracanal medicaments, sealers, and root-filling materials are also discussed.

### 1.1. Antibacterial Properties

**Figure 2:**
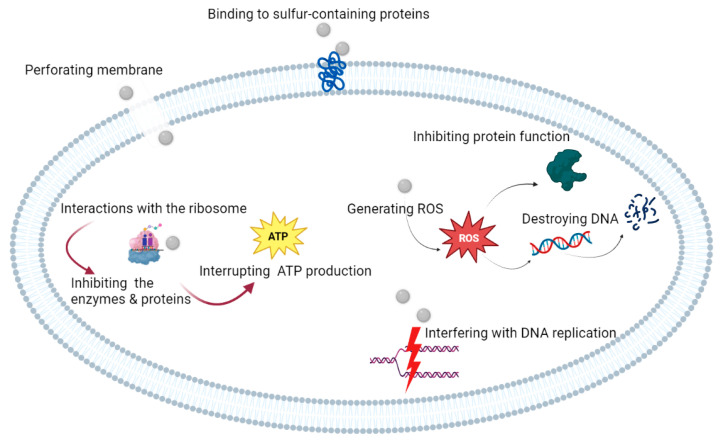
Mechanism of action of silver nanoparticles on bacteria.

The antimicrobial and cariostatic properties of silver compounds have been utilized in dentistry since the 1800s. Silver nitrate was first applied to decrease the incidence of caries in primary dentition [6]. It was later used for caries prevention in permanent molars, as a cavity sterilizing agent, and also as a desensitizing agent for dentin hypersensitivity [6]. AgNPs have optimal antibacterial properties, which depends on the concentration, type, and form of AgNPs [4]. According to Morones et al., the antibacterial properties of nanoparticles depend on their size. Nanoparticles in the range of 1–10 nm in size bind to the cell membrane and severely disrupt the membrane permeability and respiration of cells [7]. Nanoparticles with trace silver content have demonstrated strong antibacterial activity [8]. These particles may also bind to sulfur-containing proteins in the bacterial cell membrane, alter cell permeability, impair the respiratory chain, and cause eventual cell death [9]. The major antibacterial function of silver ions is through its interactions with the ribosome and subsequent inhibition of the enzymes and proteins necessary for ATP production [10] (Figure 2).

The surface charge of nanoparticles is considered to contribute to their antibacterial activity through electrostatic interactions [11], subsequently altering membrane permeability and resulting in cell death [12]. Thus, positively-charged nanoparticles have significant antimicrobial effects against all the tested bacterial species [11]. The antibacterial property of AgNPs is also attributed to the binding of nanoparticles to bacterial cell membranes, subsequently changing the membrane charge, causing its depolarization, and eventually impairing the membrane integrity. This process disrupts the basic functions of bacterial cells such as respiration, energy transfer, and nutrient transport, and eventually results in cell death. AgNPs can generate Reactive Oxygen Species (ROS), which inhibit protein function and destroy DNA while compromising the viability of bacterial cells [13].

AgNPs penetrate into bacterial cells and interact with sulfur- and phosphorus-containing groups such as in DNA, resulting in their structural damage [7]. AgNPs exhibit antibacterial efficacy on both aerobic and anaerobic bacteria by releasing silver ions. AgNPs have a much greater effect on Gram-negative than Gram-positive bacteria due to their cell membrane composition [14]. Gram-negative bacteria have a relatively thinner cell membrane and are, therefore, more susceptible to physical degradation [12]. The antimicrobial effects of AgNPs on Gram-negative bacteria depend on the silver concentration and are closely correlated with the formation of pits on the cell membrane [12]. The inhibitory effect of AgNPs on *Enterococcus faecalis* (*E. faecalis*), which is the main bacteria isolated from cases of persistent root canal infections, reinfections, and treatment failure, has been well confirmed. After treatment with AgNPs, at least 100 differentially expressed genes were detected in *E. faecalis* [15]. AgNPs inhibit the growth and proliferation of *E. faecalis* by affecting the pathways related to environmental information processing, including membrane transport, signal transfer, and metabolism of amino acids, nucleotides, and carbohydrates, as well as energy metabolism [15].

Bacteria do not develop resistance against AgNPs; therefore, AgNPs can affect a wide spectrum of bacteria [14]. Although the precise mechanism remains unknown, the ability of AgNPs to simultaneously interact with several targets within a microbial cell, including the cell membrane, DNA, enzymes, lipids, proteins, and plasmids, hinders the emergence of bacterial resistance [16].

Kishen et al. showed that combining nanoparticles with photosensitizers increased the efficacy of antimicrobial PDT. Enhanced antibacterial activity can be attributed to generation of ROS as the results of increased photosensitizer concentration, decreased risk of photosensitizer leaking out from the target cells, decreased risk of drug resistance, improved bacterial targeting due to greater interactions as the result of surface charge of the particles, further stabilization of photosensitizer, and controlled dispersion of ROS [17]. Aydin et al. combined AgNPs with toluidine blue (photosensitizer) to enhance the antibacterial efficacy of PDT as a supplement for root canal disinfection [18].

### 1.2. Antiviral Properties

AgNPs have been shown to inhibit a wide range of viruses, including those that affect the immune system (e.g., HIV), hepatitis B, influenza, herpes, and respiratory syncytial and monkey pox viruses [19]. Recent studies have recognized them as a potent and effective virucide and a new therapeutic option against viruses [20]. AgNPs can attack several targets, mediating virus proliferation. This strategy is beneficial as viruses show a slim chance of surviving through mutation or developing drug resistance [20]. They can rapidly inactivate HIV molecules after entry and during the first viral proliferation phase [20]. AgNPs also inhibit the process of fusion of a virus with host cells, since blocking HIV’s entry into its target cells can suppress viral infection, proliferation, and toxicity induced by cell–virus interactions [20]. The mechanisms of action of these nanoparticles against viruses include preventing viruses from entering the cells, preventing virus gene transcription, altering viruses’ structure, binding to glycoproteins on the virus surface, and preventing their binding to the target cells [19] without eliciting significant toxicity at low concentrations [19,20].

### 1.3. Antifungal Properties

The antifungal activity of AgNPs has been confirmed against 44 fungal strains [1]. AgNPs exert their antifungal effects against *Candida (C.) albicans* by destroying the cell membrane and preventing cell growth [21]. The minimum inhibitory concentration of AgNPs and clotrimazole and their combinations indicated synergistic activity depending on the fungal species. However, fungi may develop resistance against AgNPs. In such cases, elimination of the resistant strain would be less likely to be potentiated by AgNP–antifungal agent combination therapy [22]. It has been demonstrated that calcium hydroxide [Ca(OH)_2_] and 2% chlorhexidine gluconate (CHX) gels displayed greater antifungal effects than AgNP gel against *C. albicans* [23].

### 1.4. Anti-Inflammatory Properties

AgNPs have been suggested to promote wound healing because of their potential biological properties, such as antibacterial activity, antioxidant property [24], and anti-inflammatory effects [25]. These nanoparticles are compatible with fibroblasts and keratinocytes [26]. They also inhibited the production of proinflammatory cytokines such as IL-6, IL-1 beta, and tumor necrosis factor (TNF)-alpha should also be kept in mind [27]. Even at low concentrations, AgNPs decrease some inflammatory cytokines and angiogenic factors due to their physico-chemical characteristics [26].

### 1.5. Toxicity of AgNPs

The toxicity of AgNPs is directly correlated with their free silver ion content [28]. Due to their nano-scale dimensions, AgNPs can easily alter the normal activity of bioactive molecules, eukaryotic cells, and tissues. The type of cell response to AgNPs differs across cell types and depends on the AgNPs’ physical and chemical structure [29]. AgNP-induced toxicity due to oxidative stress generates free radicals that accumulate in the cytoplasm and cell nucleus. AgNPs possess a higher degree of early toxicity due to their high contact area, while their toxicity decreases over time as they interact with organic compounds in vivo. Application of high doses of AgNPs results in their accumulation in different organs, especially in the liver and spleen [26]. The ability of these nanoparticles to pass through the blood–brain barrier by trans-synaptic transport and accumulate in the brain is also an added concern [28,30]. At any amount, silver accumulated in organs is often cleared after 8 weeks [28]. In excessively high amounts, AgNPs have destructive effects on mitochondrial function. Application of amounts > 200 mg/kg body weight generated free radicals, released reactive oxygen species, and caused cell damage [31].

Takamiya et al. investigated the cytotoxicity of different types of AgNPs for L929 murine fibroblasts and connective tissue reactions of mice to these nanoparticles. AgNPs with an average size of 5 nm synthesized with ammonia or polyvinylpyrrolidone at a concentration of ≤25 µg/mL were not toxic and did not induce significant production of IL-1B or IL-6. When treated with 5 g/mL AgNPs, L929 murine fibroblasts released more stem cell factors after 48 h [32]. An animal study on rats reported no negative effects for orally administered AgNPs [26]. A clinical trial on the toxic effects of a commercial AgNP colloid demonstrated that oral consumption of this colloid for 14 days generated Ag+ ions in human serum but did not induce clinically significant alterations in metabolism, blood, urine, vital signs, or physical or radiographic findings [33]. Moreover, it has been shown that AgNPs are excreted in the feces and only a small amount of them is absorbed [34].

## 2. Application of AgNPs in Endodontics

Conventionally, root canal disinfection has relied on a combined mechanical instrumentation and chemicals such as sodium hypochlorite (NaOCl) to disinfect the root canal system. The presence of large degrees of uninstrumented areas within the root canal and reactive/caustic nature of NaOCl has necessitated the need for advanced antimicrobial strategies that can be effective against bacteria within these uninstrumented portions of the root canals while displaying minimal cytotoxic effects [35]. Nanoparticle-based therapeutic approaches are one such strategy that has the potential to improve the antibacterial and anti-biofilm efficacy in root canal therapy [36,37,38].

Silver is the most widely used metal nanomaterial for inhibiting several types of microorganisms and drug-resistant microorganisms [37]. Due to its significant antibacterial effects against both Gram-negative and Gram-positive pathogens, AgNP confer certain benefits in dentistry, especially for endodontic treatment [39]. AgNPs have received attention as effective disinfectants for addition to irrigants, intracanal medicaments, and root canal sealers due to their antibacterial activity against *Staphylococcus aureus (S. aureus), E. faecalis*, *Bacillus pumilus*, and *Porphyromonas gingivalis* (*P. gingivalis*) biofilms, resolution of periodontal, endodontic, or combined lesions, optimal analgesic and anti-inflammatory properties, and favorable biocompatibility. Incorporation of AgNPs in the composition of root-filling materials and mineral trioxide aggregate (MTA) has also been reported [37,40,41,42,43]. An in vitro study suggested that AgNPs enhanced or at least produced an equal effect in the resolution of root canal infection when compared with traditional disinfection methods [44].

### 2.1. Novel AgNP-Based Irrigants and Medicaments

Ertem et al. showed that a multi-purpose solution can be made to prevent biofilm regrowth in root canal infections by using porous SiO_2_-coated AgNPs in combination with several irrigating solutions. Contrary to the current treatment methods that use NaOCl in a wide range of concentrations and ethylenediaminetetraacetic acid (EDTA) sequentially, the all-in-one irrigation solution made by simple mixing of the two irrigants (NaOCl and AgNPs) with chelating agents (e.g., sodium phytate or ethylene glycol-bis N, N, N′, N′-tetraacetic acid) can be employed as a one-step irrigant to save time. Even after prolonged contact of up to 2 h, this novel solution showed lower cytotoxicity than the widely used irrigants [45].

AgNPs can be incorporated in the molecular structure of mesoporous calcium silicate nanoparticles (MCSNs). These nanoparticles can release silver ions and inhibit the growth of planktonic *E. faecalis* or biofilm formation on dentin. MCSN-Ag has the potential to become a new intracanal disinfectant owing to its antibacterial effects and low cytotoxicity. It is mainly used for the reconstruction of infected bone defects or to synthesize multifunctional biomaterials for controlled drug or bioactive molecule release systems [46]. Zheng et al. (2018) designed and evaluated a novel intracanal disinfectant in the form of glycerol monooleate-based lyotropic liquid crystal combined with CHX and AgNPs. This disinfectant penetrated deep into complex and narrow root canals and showed long-term antibacterial activity against resistant bacteria and optimal flowability [14]. Moreover, 0.02% AgNPs tailored with amorphous multi-porous bioactive glass remarkably diminished *E. faecalis* biofilm and were effective for up to 24 h after use. These antimicrobial agents can be employed as potentially effective medicaments for regenerative endodontic procedures [47]. Some related studies are listed in Table 1.

### 2.2. Effects of AgNP-Based Irrigants Compared with Conventional Irrigants

NaOCl is the current gold standard for root canal chemical disinfection [48]. The antibacterial effects of irrigants, e.g., NaOCl, CHX, and AgNPs depend on their concentration [13], contact time, and contact surface area [49]. Ioannidis et al. (2019) reported the efficacy of AgNPs synthesized on an aqueous matrix of graphene oxide (GO) in the elimination of microorganisms and biofilm in an infected tooth model. They found that ultrasonic activation of Ag-GO selectively enhanced its antimicrobial properties and biofilm disruption in lateral canals. They showed that 2.5% NaOCl disrupted maximum biofilm on dentinal tubules, while Ag-GO caused significant reductions in biovolumes compared with other experimental groups [48]. NaOCl decreased the modulus of elasticity and flexural strength of dentin, and caused toxic damage to periapical tissues, while AgNP solution did not significantly affect the mechanical properties of dentin [48]. AgNPs used as a final irrigant in root canal therapy increased the fracture resistance of endodontically treated roots [50].

Yin et al. showed that AgNPs at low concentrations were more biocompatible than NaOCl [26]. As an irrigant, AgNPs are as effective against *E. faecalis* as 2.5% NaOCl and 2% CHX, and can therefore be used as an alternative to NaOCl [51]. Likewise, other studies reported that AgNPs have strong antibacterial effects against *E. faecalis* [52,53,54]. An in vitro study by Alsamhari et al. indicated that 5.25% NaOCl and 2% liquid AgNPs were preferred for the eradication of *E. faecalis*, *S. aureus*, *Pseudomonas aeruginosa* (*P. aeruginosa*), and *C. albicans* biofilms [55]. The antimicrobial effect of AgNP solution as an irrigant in treatment of deciduous teeth was examined against monospecies *E. faecalis*, demonstrating its potential for application as an alternative to other root-canal-irrigating solutions [56]. A variety of methods are available for preparing AgNPs, including biological, physical, chemical, photochemical, electrochemical, sonolytic, radiolytic, and photochemical processes. AgNPs are best prepared using biological methods, since NPs produced in this way have a longer shelf life and stability as a result of natural capping. The main sources of AgNPs in the biosynthesis process are plant extracts, bacteria, and fungi [57]. It was also confirmed that biosynthesized AgNPs were effective on *Bacillus pumilus, P. gingivalis*, and *E. faecalis* biofilms, and hence suggested as a root canal irrigant or intracanal medicament [42]. Thus, AgNPs, with their unique chemical and physical properties, are considered an effective antimicrobial for root canal therapy [58].

Gomes-Filho et al. examined the biocompatibility and disinfection efficacy of AgNPs with 23 and 47 ppm concentrations compared with 2.5% NaOCl. It was found that the dispersed AgNPs were biocompatible and served as a disinfectant within infected dentinal tubules, especially in 23 ppm concentration [59]. AgNPs in combination with diode laser were recently introduced as a new method for bacterial disinfection. Ambalavanan et al. (2020) showed that application of AgNPs alone or combined with Nd:YAG laser irradiation was an effective protocol for elimination of resistant pathogens, such as *E. faecalis* [60]. Diode laser serves as a disinfection method in endodontics owing to its potent antibacterial effects without damaging the tooth structure or periodontal tissues. The efficacy of AgNPs in reduction of bacterial load as a less expensive method has been confirmed [61]. A combination of metal nanoparticles and diode laser was successful in decreasing *Streptococcus mutans* (*S. mutans*) microbial colonies and can be used for dentin disinfection [62]. It has been demonstrated that PDT utilizing AgNPs, a 810 nm diode laser, and indocyanine green photosensitizer could be used as an adjunct for root canal disinfection [63]. The effectiveness of AgNP irrigant can be promoted by activation with passive ultrasonic irrigation and photon-induced photoacoustic streaming to remove *E. faecalis* from the root canal system [64]. An in vitro study compared the antibacterial effects of AgNPs and gold nanoparticles with/without Nd:YAG laser irradiation against *E. faecalis* inoculated in human root dentin. They showed that irradiation using an Nd:YAG laser, along with AgNP irrigation, significantly decreased *E. faecalis* colonies compared with other groups and can, therefore, be used for root canal disinfection [65]. Evaluation of the efficacy of AgNPs, 2% CHX, and their combination against endodontic pathogens such as *E. faecalis*, *Klebsiella pneumoniae*, and *C. albicans* revealed the synergistic effect of the AgNP-CHX solution compared with each one alone [66].

A significant reduction in *Escherichia coli* (*E. coli*) count was observed following the application of 70 µg/mL AgNPs and 5.25% NaOCl as the final irrigant for the rapid disinfection of infected gutta-percha. AgNPs, at a concentration of 50 µg/mL, decreased but did not completely prevent proliferation of bacteria. However, AgNPs were highly effective against *E. coli* in 70 µg/mL concentration, and demonstrated an antimicrobial effect similar to that of 5.25% NaOCl at a 750-times-lower concentration [12]. The antibacterial effectiveness of AgNPs as a final irrigant on *E. faecalis* is similar to that of NaOCl. AgNPs can also remove the smear layer; therefore, they are suggested to be a good option for eliminating residual *E. faecalis* from root canals [67]. Furthermore, 5.25% NaOCl had the highest antibacterial efficacy, followed by AgNP irrigant and AgNPs + 17% EDTA, while the greatest smear layer removal efficacy was seen when AgNPs + 17% EDTA and 5.25% NaOCl + 17% EDTA were used as irrigants [68].

In vitro evaluation of a modification of 17% EDTA with AgNPs (EDTA-AgNPs) showed chelating and antimicrobial effects against *C. albicans* and *S. aureus* in planktonic and biofilm cultures [69]. The antimicrobial and biofilm anti-adhesion activities of AgNPs (50 µm) coated with polyvinyl alcohol (AgNPs-PVA) and 2% farnesol against *E. faecalis*, *C. albicans*, and *P. aeruginosa* were examined in a previous study. It was suggested that AgNPs-PVA and farnesol, when used after biomechanical preparation, have the potential to be applied for root canal disinfection and biofilm inhibition [70].

Contrary to the abovementioned studies, some studies have reported the superiority of conventional endodontic irrigants when compared to AgNP-based irrigants. Rodríguez-Chang et al., for example, examined the antibacterial effect of AgNPs at a concentration of 100 µg/mL with 5% NaOCl after 5 and 30 min in an in vitro study. They reported that AgNPs as an irrigant were not efficient for elimination of *E. faecalis* [71]. Sabry et al. also reported that NaOCl was the most effective antibacterial agent against *E. faecalis*, while AgNP solution was not effective as a root canal irrigant [72]. Another in vitro study by Rodrigues et al. demonstrated that 2.5% NaOCl disrupted biofilm and eliminated the bacteria in dentinal tubules and was proven to be a suitable irrigant. On the other hand, an AgNP solution of 94 ppm concentration was not effective as a root canal irrigant in eradication of *E. faecalis* biofilm and eliminating this microorganism from infected dentinal tubules [73]. Nabavizadeh et al. found that AgNP solution at 5.7 × 10^−8^ mol L^−1^ concentration effectively eliminated *E. faecalis* biofilm and did not significantly differ from 2.5% NaOCl [74]. According to an in vitro study by Kangarlou et al., AgNP solution had lower but acceptable antimicrobial activity against *E. coli*, *E. faecalis*, *P. aeruginosa*, and *C. albicans* compared with CHX and NaOCl [75].

The method of synthesis and concentration of AgNPs considerably affect their antibacterial effects. For instance, the antibacterial activity of AgNPs in 0.1% and 0.2% concentrations against five bacterial strains was similar to that of pure 0.2% CHX solution and 0.2% CHX mouthwash [76]. Furthermore, 0.1% AgNP solution for 2 min as an irrigant showed lower efficacy in eliminating residual bacterial biofilm in root canal disinfection compared with 0.01% and 0.02% AgNP gels used for 7 days as a medicament [77]. Moazami et al. suggested that AgNPs cannot be used as an intracanal irrigant due to their tooth discoloration potential [78]. This property may not be an issue in the posterior teeth [79]. It was also reported that AgNPs coated with imidazolium can cause discoloration similar to blood [78]. Studies that used AgNPs as an irrigant are summarized in Table 2.

It is worth mentioning that AgNPs can easily oxidize into silver ions when exposed to oxidizing agents, suggesting the oxidative dissolution of AgNPs. NaOCl can rapidly oxidize most AgNPs due to its powerful oxidizing ability. In addition, the interaction between NaOCl and AgNPs results in a decrease in pH [80]. Therefore, it is recommended to avoid the simultaneous use of these two irrigants.

### 2.3. Effect of AgNP-Based Medicaments Compared with Conventional Medicaments

Nanoparticles enhance the effectiveness of intracanal medicaments, and longer contact time further enhances their antimicrobial properties [81]. Therefore, prolonged exposure time of bacteria to intracanal medicaments can significantly eliminate the biofilms [82,83]. AgNPs as medicaments are effective against several drug-resistant bacteria, and therefore can be used to treat a wide variety of infections [84]. Due to their small size, AgNPs can eliminate bacteria from hard-to-reach areas not accessible by other drugs [85]. In drug delivery systems, AgNPs can enhance drug solubility, stability, and bio-distribution. Drug absorption increases in the presence of nanoparticles; therefore, AgNPs can be used in drug delivery systems [84]. As an intracanal medicament with antibacterial effects, AgNPs can gradually increase the dentin micro-hardness of endodontically treated teeth over time and can, therefore, be an alternative to Ca(OH)_2_, which has a destructive effect on dentin microhardness [86].

Some studies have confirmed the enhanced effect of conventional intracanal medicaments when combined with AgNPs as carrier. Javidi et al. introduced a combination of Ca(OH)_2_ and AgNPs as a medicament that significantly decreased the intracanal *E. faecalis* count [87]. Afkhami et al. examined several single-rooted teeth infected with *E. faecalis* and exposed to different intracanal medicaments, including Ca(OH)_2_ with saline, Ca(OH)_2_ with CHX, Ca(OH)_2_ with AgNP suspension, and saline as the control group. The results indicated that AgNPs were more effective on *E. faecalis* biofilm compared with other tested carriers in the short term [88]. A combination of AgNPs with Ca(OH)_2_ was shown to be more effective in eliminating the bacteria from the root canals and demonstrated greater anti-inflammatory and antioxidant effects [89,90]. Antibacterial evaluation of silver and cadmium (Cd) nanoparticles and Ca(OH)_2_ against *E. faecalis* biofilm showed that AgNPs medicament was more effective than CdNPs, whereas Ca(OH)_2_ was not effective against *E. faecalis* biofilm [91]. Poloxamer-based thermoreversible gel of AgNPs showed prolonged release of Ag^+^ and strong anti-biofilm properties against *E. faecalis* for 9 days. At 16 μg/mL and 32 μg/mL concentrations, it was clinically beneficial for the eradication of *E. faecalis* biofilm on dentin and within dentinal tubules [92]. Bruniera et al. demonstrated that AgNPs combined with carriers such as Carbomer and polyethylene glycol, especially hydroxyethylcellulose, formed stable formulations. Therefore, AgNPs are potential root canal disinfectants that have wider applications when combined with carriers [93]. Nevertheless, discoloration is a problem associated with the application of AgNPs in anterior teeth [94].

Afkhami et al. reported that AgNPs added to Ca(OH)_2_ paste did not cause significant tooth discoloration compared with Ca(OH)_2_ alone, and prolonged use of AgNPs/Ca(OH)_2_ for 3 months did not increase discoloration. As noted, the application of AgNPs must be limited to the root canal space, and any residues in the pulp chamber must be carefully removed before restoring the crown [95]. Although many studies reported the positive effects of using AgNPs, some others did not report a remarkable efficacy for AgNPs as an intracanal medicament or showed an efficacy comparable to other endodontic medicaments. In vivo studies by Chandra et al. indicated greater antimicrobial effect of 2% CHX as an intracanal medicament compared with AgNPs and Ca(OH)_2_ on *E. faecalis* and *C. albicans* biofilm at 24 h, 7 days, and 14 days. Adding nanoparticles to this medicament did not improve its antibacterial effects [96,97]. Another study concluded that AgNPs were less effective against *E. faecalis* than Ca(OH)_2_ alone or with AgNPs [98]. Ca(OH)_2_ combined with AgNPs decreased the bacterial count at 1 and 2 weeks, the reduction in bacterial count was greater when Ca(OH)_2_ was used alone [98]. AgNPs in Plectrantus ambionicus extract were less effective than Ca(OH)_2_ against *E. faecalis* and *C. albicans* [99]. Mozayeni et al. confirmed the greater antifungal activity of Ca(OH)_2_ and 2% CHX compared to AgNP gel on *C. albicans* [23]. Some studies have also reported the failure of AgNPs in root canal therapy, especially in the long term. For example, Salas-Orozco et al. reported a higher prevalence of resistant genes to AgNPs in endodontic pathogens in the long term. This highlights the need for re-evaluation of the application of nanoparticles (especially AgNPs) as an antimicrobial medicament in endodontics. The development of resistant genes can have serious side effects, such as increased resistance to antibiotics and other antimicrobial agents and can even complicate treatment of persistent infections (e.g., secondary endodontic infections) [100]. Studies that used AgNPs as a medicament are summarized in Table 3.

### 2.4. Effect of AgNP-Based Sealers and Root-Filling Materials Compared with Traditional Root-Canal-Filling Materials

The antibacterial activity of the commonly used sealers often lasts for a maximum of one week; following this period, its antibacterial properties decline markedly. Long-term antibacterial activity of root canal sealers would be highly useful for more efficient root canal disinfection; thus, adding antibacterial nanoparticles to root-canal-filling materials can improve direct and sustained antibacterial effects [17]. Incorporation of AgNPs in the composition of sealers enhances their flowability; among different nanoparticles used for this purpose, AgNPs combined with sealers had the greatest penetration depth into dentinal tubules due to the small size of AgNPs [101]. The addition of AgNPs to conventional root canal sealer (powder) markedly improved their antibacterial properties [102]. Farahat et al., in an in vitro study, indicated that the addition of AgNPs to AD Seal, MTA Fillapex and GuttaFlow 2 increased their antibacterial activity [103]. Aristizabal et al. evaluated the antimicrobial efficacy of mixing AgNPs with zinc oxide eugenol cement against *E. faecalis*. The results demonstrated the antibacterial activity of AgNPs against *E. faecalis* and a significant difference between nanoparticles suspended in guava extract and other groups [104]. The addition of AgNPs to zinc polycarboxylate cement can improve the density and antimicrobial activity of this endodontic cement against *E. coli*, *S. aureus*, and *C. albicans* [105].

The new generation of bioactive root canal sealers combined with bioactive additives exhibit antibacterial and remineralizing properties [106]. Incorporating additives such as quaternary ammonium methacrylate and AgNPs into novel bioactive and therapeutic root canal sealers resulted in a reduction in biofilm CFU by six logs while having a minimum negative effect on physical and sealing properties. By using newly developed, therapeutic, bioactive materials, root canal procedures can be performed more efficiently, and tooth survival can be increased [106].

Baras et al. introduced a new endodontic sealer containing dual-cure methacrylate with a mass ratio of 5% dimethylaminohexadecyl methacrylate, 0.15% AgNPs, and 10%, 20%, and 30% amorphous calcium phosphate nanoparticles. This new sealer targeted the residual bacteria and guaranteed primary treatment success. In case of future microleakage, this sealer can prevent secondary infections by releasing a high level of calcium and phosphate ions and fortifying and protecting the root structure [107]. Baras et al. formulated a biological bioactive sealer containing dual antibacterial dimethylaminohexadecyl methacrylate with 0%, 2.5%, and 3% mass percentages, and AgNPs with 0.05%, 0.1%, and 0.15% weight percentages. This sealer showed strong anti-biofilm activity without compromising its physical and sealing properties. The combination of dimethylaminohexadecyl methacrylate and AgNPs in this sealer decreased biofilm survival. This new sealer has two main benefits: antimicrobial properties, and prevention of secondary infection and re-infection of the root canal system [108].

Some studies reported no superiority of AgNP-based sealers. Recently, Afkhami et al. (2021) showed that a combination of AgNPs and AH Plus sealer did not prevent bacterial leakage [109]. Likewise, Haghgoo et al. showed that adding AgNPs up to 5 wt% did not improve the antibacterial properties of zinc oxide eugenol sealer [110].

AgNP coating of gutta-percha cone was also evaluated for antibacterial activity [20]. This new material (standard gutta-percha with a thin coating of AgNPs) had significant effects against *S. aureus*, *C. albicans*, and *E. coli*. Its biocompatibility was examined by comparing the cytotoxicity caused by this new material and the standard gutta-percha against murine fibroblasts. At 24 h, the cytotoxicity of gutta-percha with AgNP coating was similar to that of standard gutta-percha. However, this value was decreased significantly after 1 week [20,111]. AgNP-coated gutta-percha was observed to be more effective in preventing microleakage than the standard gutta-percha in obturated root canals [112]. Coating of gutta-percha with AgNPs and chitosan in 1% and 2% concentrations demonstrated concentration-dependent antibacterial activity for both gutta-percha forms; coating with AgNPs resulted in higher antibacterial activity compared with gutta-percha coated with chitosan nanoparticles [113]. Another study concluded that gutta-percha coated with AgNPs possessed both antibacterial and antifungal properties [111], in addition to preventing bacterial leakage similar to standard gutta-percha [112]. No difference was observed in the in vitro cytotoxicity and in vivo subcutaneous tissue inflammation between the two gutta-percha groups. In a biocompatibility study on a rat model, gutta-percha coated with AgNPs was biocompatible and acceptable for root canal obturation [114]. Studies utilizing AgNPs as a root-filling material are summarized in Table 4.

### 2.5. Effect of Addition of AgNPs to MTA

Mixing MTA with AgNPs enhanced antibacterial activity against anaerobic endodontic–periodontal pathogens, e.g., *E. faecalis* and *P. aeruginosa*, and improved antifungal activity against *C. albicans* [115]. According to Afkhami et al., AgNPs combined with MTA can be used as an orifice plug to prevent bacterial leakage in endodontically treated teeth [116]. Due to low radiopacity, MTA cannot be well visualized radiographically; an addition of 1 wt% AgNPs improves MTA radiopacity [117]. Additionally, the addition of AgNPs to calcium silicate cements can increase their pH and compressive strength and enhance their radiopacity and setting time [118]. MTA incorporated with AgNPs has good biocompatibility and does not induce an inflammatory response [119,120]. However, it does not have any significant positive effect on bio-mineralization properties of MTA either [121]. On the other hand, an in vitro study showed that application of Ca(OH)_2_/AgNPs as an intracanal medicament after 1 week or 1 month had no significant effect on bond strength of MTA to root dentin [122]. Another therapeutic application of AgNPs is the mixing of MTA with >6% colloidal solution of 0.1 mg/mL AgNPs instead of water, which promotes antimicrobial activity against *Fusobacterium nucleatum* [123]. Furthermore, mixing MTA with >12% colloidal solution of 0.1 mg/mL AgNPs instead of water increases its antimicrobial activity against *P. gingivalis* [124]. AgNPs added to MTA and calcium-enriched mixture cement at low concentrations can increase their antimicrobial properties [125]. If these results are confirmed in vivo, such mixtures may find potential application in the treatment of root perforation repair [124]. Studies that added AgNPs to MTA are summarized in Table 5.

### 2.6. Effect of Addition of AgNPs to Fiber Posts

Some studies have evaluated the application of AgNPs combined with commonly used root canal fiber posts. Much attention has been paid to the use of optical fibers with AgNPs due to their bacteriostatic properties as a root canal filler. The coating of optical glass fiber posts with a thin layer of AgNPs markedly increases the optical fibers’ hardness, modulus of elasticity, and resistance. Light transfer through the optical fiber structure, which makes it possible to photo-cure the fluid resins in the canal, and optimal compatibility with resin cements and glass fiber posts are among the other advantages of AgNP coating of posts [126]. Poggio et al. studied the antimicrobial properties of a new fiber post with incorporated AgNPs and reported a fair antibacterial activity against *S. mutans*, *Streptococcus salivarius*, and *Streptococcus sanguis*. In addition to high biocompatibility, it decreased the occurrence of secondary caries and enhanced the survival of tooth-restoration complex [127].

### 2.7. Application of AgNPs in Endodontic Surgery

The hydraulic properties of tricalcium silicate-based cements enable their use as root-end filling materials in part due to their setting even in presence of blood and tissue fluids. The biocompatibility and sealing ability of calcium silicate cements such as MTA have also shown promising results. Bioactivity is another notable characteristic which affects the surrounding tissues [128].

The addition of calcium chloride (an accelerant) and AgNPs to calcium silicate-based cements resulted in favorable physicochemical properties such as higher initial pH, release of calcium ions, and optimal dimensional stability. Such factors contribute to a germ-free environment and enhance healing, which are important in endodontic surgery [128]. Silver nanoparticles have been proven to be biocompatible, particularly at low concentrations [129].

An inflammatory response in subcutaneous tissue was not elicited by the addition of 1% AgNPs to MTA in rats [130]. MTA mixed with AgNPs or titanium dioxide nanoparticles is as biocompatible as MTA alone. Therefore, AgNPs can be used as additives to enhance the antimicrobial efficacy of MTA [131]. Gold or silver nanoparticles have no effect on the overall biocompatibility of calcium silicate-based cements [132].

The MCSNs were synthesized and introduced as novel root-canal-filling materials due to their unique nanostructure, injectability, apatite mineralization, and potential drug delivery. Nano-sized MCSNs continuously release calcium and silicon ions and create a weakly alkaline environment that prevents bacterial growth. In addition, they can induce bone regeneration and defect healing. However, MCSNs have limited antibacterial activity. By adding AgNPs to MCSNs, biofilm formation will be prevented or decreased without affecting their mechanical properties. Additionally, Ag and Zn might act synergistically as antibacterial elements against *E. faecalis* and its biofilm. By adjusting the ratio of nanosilver and nanozinc in Ag/Zn-MCSNs, a good balance between antibacterial activity and cytotoxicity can be achieved. They eliminate bacteria by releasing Ag, which destroys the cell membrane [133]. A future bone cement can be synthesized by adding AgNPs to Portland cement, which has acceptable mechanical strength, biodegradability, and biocompatibility. However, in vitro and in vivo investigations, as well as long-term studies, are required to clarify the additional benefits of using AgNPs in different clinical settings [134].

### 2.8. Effect of AgNPs on Postoperative Pain

A study on the efficacy of AgNPs as an intracanal medicament to mitigate postoperative pain in necrotic teeth with apical periodontitis after 4, 12, and 24 h showed that they were significantly more effective than Ca(OH)_2_; however, no significant difference was observed after 48 h [135]. Another randomized controlled clinical trial evaluated the effect of using Ca(OH)_2_/AgNPs (0.03 μg/mL concentration) in 2:1 ratio or Ca(OH)_2_ individually as an intracanal medicament in reducing postoperative pain and intracanal bacterial count, and showed that Ca(OH)_2_/AgNPs decreased the count of intracanal bacteria more than each one alone, although this difference was not significant for aerobic intracanal bacteria. On the other hand, it decreased the incidence and severity of inter-appointment pain. Resultantly, this combination should be tested in different concentrations with different ratios to find the most effective combination with potential applications in endodontic therapy [136]. The positive impact of AgNPs and nano-Ca(OH)_2_ intracanal medicaments on post-endodontic pain and flare-ups in retreatment cases was recently demonstrated; however, the antibacterial effect of AgNPs was comparable to that of Ca(OH)_2_ [137].

## 3. Conclusions and Prospects

This review focused on the application of AgNPs in contemporary root canal procedures. The antibacterial effects of AgNPs against intracanal pathogens have been widely confirmed through predominantly in vitro investigations. The antibacterial properties of AgNPs will depend on the method of synthesis, concentration, type, and form employed for different applications. The application of low concentrations of AgNPs in endodontics resulted in significantly less cytotoxicity compared to NaOCl, in addition to demonstrating no untoward effects on the mechanical integrity of root dentin. At low concentrations AgNPs are more effective as a medication compared to an irrigant. Additional studies are warranted to determine the ideal concentration of AgNPs to ensure optimum antimicrobial effects without cytotoxicity in vivo. With respect to the synergistic effect of antibacterial properties of AgNPs in combination with the commonly used medicaments as well as sealers, novel compositions based on these nanoparticles should be developed for safe and effective root canal therapy. Further studies are also required to investigate the tooth discoloration potential of AgNPs.

## Figures and Tables

**Table 1:** Studies of alternative antimicrobial strategies highlighting antimicrobial type, study design, usage, experimental and control groups, microorganisms tested, and main findings.

Author	Study Design	Usage	Experimental Groups	Control Group	Microorganism	Main Findings
Ertem et al., 2017 [45]	Human root model	Irrigant	0.18 mM AgNPs@SiO_2_ + 0.75 mM Tris + 3% (*w*/*w*) NaOCl + 35% (*w*/*w*) SP, 0.18 mM AgNPs@SiO_2_ + 0.75 mM Tris + 3% (*w*/*w*) NaOCl + 35% (*w*/*w*) EGTA in UPW	Untreated biofilms	*Fusobacterium nucleatum * *Actinomyces naeslundii * *E. faecalis * *Streptococcus sanguinis Streptococcus sobrinus*	In comparison with classically used solutions, AgNPs/SiO_2_-containing solutions have shown to be less cytotoxic. Biomedical devices may benefit from this proactive long-term disinfection approach based on nanomaterials.
Fan et al., 2014 [46]	Human root model	Irrigant	Mesoporous calcium-silicate (MCSNs) Ag-MCSNs-A, AgNPs -incorporated MCSNs prepared by the adsorption method Ag-MCSNs-T, AgNPs -incorporated MCSNs prepared by the template method	Bacteria inoculum without nanoparticles	*E. faecalis*	In planktonic or colonized forms, Ag-MCSNs-T showed similar antibacterial effects to Ag-MCSNs-A but were significantly less toxic.
Zheng et al., 2018 [14]	Human root model	Medicament	Glycerol monooleate (GMO) LLC precursor incorporation with chlorhexidine (CHX) and AgNPs GMO–ethanol–water (48%: 12%: 40%, *w*/*w*)	Ca(OH)_2_	*E. faecalis*	In comparison with Ca(OH)_2_, cubic precursors incorporated with 0.5% CHX and 0.02% AgNPs showed a significant increase in antibacterial activity against *E. faecalis*

**Table 2:** Studies of alternative antimicrobial irrigants highlighting antimicrobial type, study design, usage, experimental and control groups, microorganisms tested, and main findings.

Author	Study Design	Usage	Experimental Groups	Control Group	Microorganism	Main Findings
Ioannidis et al., 2019 [48]	Human root model	Irrigant	Aqueous suspension of 0.25% Ag-GO, 1% and 2.5% NaOCl, 2% CHX, 17% EDTA	Sterile saline	*Propionibacterium acnes * *Actinomyces radicidentis Staphylococcus epidermidis * *Streptococcus mitis * *E. faecalis*	All sampling sites showed superior antimicrobial efficacy with NaOCl 2.5% and the least affected area was found to be the middle root third lateral canal.
AL-Fahham et al., 2019 [51]	Human root model	Irrigant	AgNPs, NaOCl CHX	Normal saline	*E. faecalis*	Using AgNPs as irrigation solutions can effectively remove *E. faecalis* biofilms similar to sodium hypochlorite.
Moradi et al., 2018 [56]	Human root model	Irrigant	AgNPs solution, NaOCl	Normal Saline	*E. faecalis*	Other root canal irrigants can be replaced with AgNPs solution.
Halkai et al., 2018 [42]	Human dentin block model	Irrigant	AgNPs, 2% and 0.2% CHX	Distilled water	*Porphyromonas gingivalis * *Bacillus pumilus* *E. faecalis*	Endoperio pathogens are susceptible to fungal-derived AgNPs.
Makkar et al., 2018 [58]	Brain Heart infusion agar plate	Irrigant	Combination of AgNPs ethanol and NaOCl	NaOCl 3%	*E. faecalis * *S.aureus * *C. albicans*	It is effective to use AgNPs based irrigant for endodontic treatment.
Gomes-filho et al., 2013 [59]	Wistar albino rats received infected or uninfected tubes	Irrigant	AgNPs dispersion (23 and 47 ppm) 2.5% NaOCl	Saline solution	*-*	Especially at 23 ppm, AgNPs dispersion may be able to act as disinfectants in contaminated tubes.
Ambalavanan et al., 2020 [60]	Trypticase soy agar plates.	Irrigant	AgNPs in combination with or without Nd-YAG laser	No treatment	*E. faecalis*	The use of AgNPs alone or in conjunction with Nd: YAG laser irradiation would be effective against *E. faecalis.*
Sadony et al., 2019 [61]	Human root model	Irrigant	AgNPs diode laser	No treatment	*E. faecalis*	The antibacterial properties of diode lasers allow them to be used as adjunctive endodontic disinfection modalities.
Alsamhari et al., 2022 [55]	Tissue culture method/microtiter plate method	Irrigant	2.5%, 5.25% NaOCl, 2.0% CHX liquid and 60 mg/L AgNPs	Sterile saline	*E. faecalis,* *S. aureus* *Pseudomonas aeruginosa * *C. albicans*	5.25% NaOCl and 60mg/L AgNPs liquid are preferred for removing biofilm microorganisms from liquid supplies.
Rajasekhar et al., 2022 [68]	Human root model	Irrigant	AgNp, AgNPs + 17% EDTA 5.25% NaOCl, NaOCl 5.25% +17% EDTA	Distilled water	*E. faecalis*	The most effective antibacterial irrigant is 5.25% NaOCl, followed by AgNps and AgNPs + 17% EDTA. When AgNPs + 17% EDTA irrigant and 5.25% NaOCl + 17% EDTA irrigant were used, the greatest smear layer removal efficacy was seen.

**Table 3:** Studies of alternative antimicrobial medicament highlighting antimicrobial type, study design, usage, experimental and control groups, microorganisms tested, and main findings.

Author	Study Design	Usage	Experimental Groups	Control Groups	Microorganism	Main Findings
Afkhami et al., 2015 [88]	Human root model	Medicament	Ca(OH)_2_/normal saline Ca(OH)_2_/CHX, Ca(OH)_2_/AgNPs suspension	Saline	*E. faecalis*	For short term treatment, Ca(OH)_2_/AgNPs were more effective than other tested vehicles against *E. faecalis* biofilms.
Javidi et al., 2013 [87]	Human root model	Medicament	Ca(OH)_2_ with or without a AgNPs suspension	Sterile water	*E. faecalis*	A combination of Ca(OH)_2_ and AgNPs significantly reduced the number of intracanal *E. faecalis.*
Chandra et al., 2017 [97]	Human root model	Medicament	Ca(OH)_2_ 2% CHX AgNPs AgNPs with Ca(OH)_2_ AgNPs with 2% CHX	Saline	*E. faecalis * *C. albicans*	In both short- and long-term studies, 2% CHX was more effective as other intracanal medicaments against *E. faecalis* and *C. albicans* biofilms.
Mozayeni et al., 2015 [23]	Human root model	Medicament	Ca(OH)_2_ CHX	Saline	*C. albicans*	The antifungal activity of Ca(OH)_2_ and 2% CHX gels are significantly higher than AgNPs gel.
Elkillany et al., 2022 [81]	Human root model	Medicament	CaOH_2_, CaOH_2_ nanoparticles, CHX, CHX loaded by AgNPs, CHX loaded by chitosan nanoparticles	No medicament	*E. faecalis*	There was a reduction in bacterial counts with all tested medicaments. Medicaments that were nanosized were more effective than normal sized.
Raza et al., 2022 [89]	Human root model	Medicament	Ca(OH)_2_ impregnated with 0.1% by weight AgNPs	unmodified Ca(OH)_2_	*E. faecalis*	Ca(OH)_2_ impregnated with AgNPs showed improved ability to eliminate biofilms of *E. faecalis.*
Arora et al., 2021 [91]	Standard size dentin sections	Medicament	Ca(OH)_2_ AgNPs gels CdNPs gels	No treatment	*E. faecalis*	Both AgNPs gel and CdNPs gel eliminated *E. faecalis* biofilms during root canal disinfection and can be used as a medicament.

**Table 4:** Studies of alternative antimicrobial filling materials highlighting antimicrobial type, study design, usage, experimental and control groups, microorganisms tested, and main findings.

Author	Study Design	Usage	Experimental Groups	Control Groups	Microorganism	Main Findings
Alzaidy et al., 2018 [102]	Agar diffusion brain-heart infusion	Sealer	0.5%, 1%, 2% and 4% additive of AgNPs particles to the weighted powder	AgNPs free	*E. faecalis*	Antimicrobial activity of the root-canal sealer increased significantly by adding AgNPs to the powder of the root canal sealer.
Baras et al., 2019 [107]	Human dentin block	Sealer	Dimethylaminohexadecyl methacrylate (DMAHDM) + AgNPs DMAHDM + AgNPs + 10NACP DMAHDM + AgNPs + 20NACP DMAHDM + AgNPs + 30NACP	AH Plus	*E. faecalis*	Endodontic therapy and tooth root strengthening can both be improved by the use of this new sealer with highly desirable antibacterial and remineralization properties.
Baras et al., 2019 [108]	linear dye penetration method Colony-forming units (CFU), live/dead assay, polysaccharide production of biofilms grown on sealers	Sealer	DMAHDM and AgNPs each alone and in combination using DMAHDM mass fractions of 0%, 2.5% and 5%, and AgNPs mass fractions of 0.05%, 0.1% and 0.15%	AH Plus	*E. faecalis*	As compared to AH Plus and experimental controls, the sealer containing 5% DMAHDM and 0.15% AgNPs significantly reduced biofilm polysaccharide production and decreased CFU.
Haghgoo et al., 2017 [110]	Disk Diffusion Test	Sealer	0, 0.5, 2, and 5 wt% AgNPs in conjunction with zinc oxide eugenol (ZOE)	N/A	*E. faecalis*	Adding AgNPs to ZOE sealer up to 5 wt% would not improve its antibacterial properties against *E. faecalis.*
Farahat et al., 2022 [103]	Brain Heart Infusion broth	Sealer	MTA Fillapex MF-AgNPs GuttaFlow 2 GF-AgNPs AD Seal AD-AgNPs	Sealer and culture media and saline solution Culture media and bacterial suspension without any sealer	*E. faecalis*	Antibacterial activity of sealers was enhanced by adding AgNPs.
Emad et al., 2022 [105]	Agar diffusion and broth dilution	Sealer	AgNPs-zinc polycarboxylate cement (ZPCCEM)	N/A	*E. coli * *S. aureus * *C. albicans*	In order to enhance ZPCCEM’s antimicrobial activity, AgNPs can be added in small amounts.

**Table 5:** Studies of alternative MTA highlighting study design, experimental and control groups, microorganisms tested, and main findings.

Author	Study Design	Experimental Groups	Control Groups	Microorganism	Main Findings
Samiei et al., 2013 [115]	Agar diffusion	MTA MTA/AgNPs 1% weight	Control plates without adding any materials	*E. faecalis* *Pseudomonas aeruginosa* *S. aureus* *C. albicans*	MTA’s antimicrobial efficacy was improved by adding AgNPs.
Nasri et al., 2021 [116]	Human root model	MTA Ag-MTA	The entire root surfaces were covered with two layers of nail varnish Root canals were filled with a single gutta-percha cone without a sealer and no orifice plug	*C. albicans, * *S. aureus * *Streptococcus mutans, * *E. faecalis * *E. coli* *Streptococcus sanguinis*	The Gray ProRoot MTA modified by AgNPs has the potential to be used in endodontic treatment as an orifice plug.
Bahador et al., 2015 [120]	Agar diffusion membrane-enclosed immersion	MTA AgNPs-MTA	1 mL of the bacterial suspension in wells not containing MTA or AgNPs-MTA Control wells were treated identically, except for Bacterial inoculation	*Aggregatibacter* *actinomycetemcomitans Fusobacterium nucleatum* *Porphyromonas gingivalis* *Prevotella intermedia*	AgNPs can be used as an excellent additive for MTA against anaerobic endodontic–periodontal bacteria with a clinical application for infection control in endodontics
Bahador et al., 2013 [123]	Agar diffusion broth dilution	MTA AgNPs-MTA	1 mL of Bacterial suspension in a well free of MTA and AgNPs-MTA A well without *F. Nucleatum*	*Fusobacterium nucleatum*	In dose dependent manner, AgNPs-MTA were found to completely inhibit the proliferation of *F. nucleatum* that may affect root perforation prognosis.
Bahador et al., 2013 [124]	Agar diffusion broth dilution	IMTA AgNPs-MTA	1 mL of bacterial suspension in a well free of MTA and AgNPs-MTA A well without *P. gingivalis*	*Porphyromonas gingivalis*	A dose-dependent effect of AgNPs -MTA on gingival proliferation may have a significant impact on root perforation prognosis.

## Data Availability

Not applicable.
